# Inter-Day Variability of Metabolites of DEHP and DnBP in Human Urine—Comparability of the Results of Longitudinal Studies with a Cross-Sectional Study

**DOI:** 10.3390/ijerph16061029

**Published:** 2019-03-21

**Authors:** Sibylle Hildenbrand, Thomas Gabrio, Gerhard Volland

**Affiliations:** 1 Institute of Occupational and Social Medicine and Health Services Research, University Hospital Tübingen, Wilhelmstrasse 27, 72074 Tübingen, Germany; 2 State Health Office of Baden-Württemberg, Nordbahnhofstrasse 135, 70191 Stuttgart, Germany; thomas-gabrio@t-online.de; 3 Material Testing Institute (MPA)—Otto-Graf-Institute, Pfaffenwaldring 32, 70569 Stuttgart, Germany; gerhard.volland@web.de

**Keywords:** human biomonitoring, di(2-ethylhexyl) phthalate (DEHP), di-*n*-butyl phthalate (DnBP), phthalate metabolites, urine, inter-day variability, comparison longitudinal studies with cross-sectional studies

## Abstract

In the current paper, we compare the inter-day variability of the metabolite concentration of di(2-ethylhexyl) phthalate (DEHP) and di-*n*-butyl phthalate (DnBP) in 247 morning urine samples obtained from 19 probands of different age and sex with the metabolite concentration in morning urine obtained from 215 probands of the “Tübingen Survey” cross-sectional study. In the first longitudinal study the morning urine of seven volunteers was collected four times a year for seven consecutive days (course of the year study). In a second study the morning urine of 12 students of a boarding school was collected on five consecutive days (course of a week study). For participants of the two different longitudinal studies we obtained mean concentrations in first void morning urine for mono(2-ethyl-5-hydroxyhexyl) phthalate (5OH-MEHP) in the range from 21.3 to 110 µg/L, 10.5 to 35.6 µg/L for mono(2-ethyl-5-oxohexyl) phthalate (5oxo-MEHP), and 45.5 to 143 µg/L for mono(2-ethyl-5-carboxypentyl) phthalate (5cx-MEPP). The corresponding relative standard deviations (rel. Std.D in %) for these DEHP-metabolites vary between 45.2% and 262%. The 50th percentiles vary for 5OH-MEHP between 17.5 and 65.6 µg/L, for 5oxo-MEHP between 9.0 and 20.3 µg/L and for 5cx-MEPP between 42.5 and 82.0 µg/L. For participants of the “Tübingen Survey” cross-sectional study the means vary for 5OH-MEHP between 58.2 and 85.0 µg/L, between 33.6 and 38.7 µg/L for 5oxo-MEHP and between 110 and 158 µg/L for 5cx-MEPP with rel. standard deviations in a range between 86.5 to 175%. The corresponding 50th percentiles vary for 5OH-MEHP between 26.5 and 42.3 µg/L, for 5oxo-MEHP between 18.0 and 26.3 µg/L, and for 5cx-MEPP between 57.2 and 77.6 µg/L. In order to compare the data from the longitudinal studies with the data from the cross-sectional study, the frequency distribution of the results of both types of studies was compared first. In a second step, the results of a *t*-test (*p*-values) was used to check whether the results of the long-term studies differ statistically significantly from the results of the cross-sectional study (*p* < 0.05). The present data show that the frequency distributions of DEHP-metabolites are comparable. For most of the participants respectively subject groups *t*-test results prove that no statistical significant difference between results obtained from longitudinal studies compared to the results of the cross-sectional study are apparent. The available data on the exposure of individual subjects mirror the data obtained from cross-sectional studies of the general population and give hints to the risk of individual increased DEHP exposure. Results also highlight the importance of living conditions on the risk of increased DEHP exposure.

## 1. Introduction

The group of endocrine substances has long played a prominent role in the risk discussion on the effects of environmental pollutants on human health. Within this group diesters of orthophthalic acid (1,2-benzenedicarboxylic acid), in particular di(2-ethylhexyl) phthalate (DEHP), dibutyl phthalate (DBP), butylbenzyl phthalate (BBzP) and diisobutyl phthalate (DIBP) are considered critical due to their high production volumes and widespread use in the manufacture of consumer goods [1,2,3]. Additionally, other phthalates like DEHP and di-*n*-butyl phthalate (DnBP) have anti-androgenic effects in animal experiments. DEHP and MnBP “are so called endocrine disruptors [which] interfere with the complexly regulated hormonal processes that control sexual differentiation [2]”. Based on these risk assessments, the use of phthalates has been subject to regulation. Annex XVII to regulation 1907/2006 (REACH) restricts the use of DEHP, DBP and BBzP in toys and childcare products [4]. Regulation (EC) No. 1272/2008 on the classification, labelling and packaging of substances and mixtures [5] classifies DEHP as a reproductive toxic substance of category 1B. Following this decision, the remaining diesters were also classified as reproduction toxic substances (category 1B) in July 2017. Moreover, because of their endocrine disrupting effects, all were included in the list of substances of very high concern (article 57(f)), and added to Annex IV according to Article 59 (10) of the REACH regulation [6]. This implies that future uses of these four orthophthalates (DEHP, DBP, BBP and DIBP) on the EU market are limited to applications for which a case-specific authorization has been granted. These legal restrictions thus limit the oral and dermal intake of these mobile phthalates via the most important vectors of exposure.

These orthophthalates have a substantial migration potential and can be present in relatively high concentrations in many of the respective products [7,8,9,10], a remarkable yet highly variable exposure of the general population to them can be observed. Due to the substantial number of articles on the subject, it is referred to a number of selected publications [11,12,13,14,15,16,17,18,19,20].

A question that arises from the high inter-individual heterogeneity in exposure to DEHP is whether similar variations can be observed intra-individually over the course of time. Seminal investigations on the issue by Calafat [15], Fromme et al. [17], Hildenbrand et al. [18] and Preau et al. [19] show that inter-day exposure of subjects can likewise fluctuate substantially over time. Their results indicate that a significant proportion of exposure to DEHP is due to sources that are encountered sporadically rather than on a regular basis. Studies assessing the relative importance of food consumption and the use of consumer goods for exposure have generally found that, with the exception of small children, a majority of DEHP intake stems from food [21,22,23].

In the present paper, we study inter-day variability in daily DEHP and DnBP exposure among different groups of subjects, and compare these findings to results of our conventional cross-sectional survey. For studying inter-day variability we analyzed first morning urine voids of seven subjects aged 19 to 58 from Southwestern Germany. These individuals were tracked for 4 × 7 days each. Additionally, we examine 60 morning urine samples collected for five consecutive weekdays from 12 boarding school students aged 17–20. The students lived in a boarding school also located in Southwestern Germany. Aside from insights into the temporal pattern of DEHP and DnBP intake for individual subjects, and the influence of environmental conditions e.g., boarding school, we are thus able to derive information on the comparability of longitudinal and cross-sectional results.

## 2. Materials and Methods

The present paper shows the results of concentration levels of mono-*n*-butyl phthalate (MnBP), mono(2-ethylhexyl) phthalate (MEHP), mono(2-ethyl-5-hydroxyhexyl) phthalate (5OH-MEHP), mono(2-ethyl-5-oxohexyl) phthalate (5oxo-MEHP) and mono(2-ethyl-5-carboxypentyl) phthalate (5cx-MEPP) in morning urine (first morning void) for different subject groups obtained through either longitudinal or cross-sectional studies. In detail:

- Longitudinal 4 × 7 days study (“course of the year“): For this study first morning voids from seven male and female subjects were collected for seven consecutive days (Monday to Sunday) in the months January, April, July and October (and thus in the four seasons of the year) in total 187 urine samples. Sampling was carried out throughout the year 2005. Three test persons aged between 19 and 28 years (two male, one female) and four subjects (two men and two women) being 40 years or older. All participants in this study were employees from the three participating research institutes or their family members.
- Longitudinal 1 × 5 days study (“course of the week“): For this study 60 morning urine samples were collected from 12 boarding school students of both sexes aged between 17 and 20 years. The participants (students) were proposed in cooperation with the school management. The urine samples of each participant were sampled on five consecutive weekdays (Monday to Friday). They provided in total 60 urine samples in December 2005. The meals of this boarding school were prepared and delivered by a cantina kitchen. Additionally, at the time of examination, buying from shops outside the school compound was possible for the students of this group.
- The cross-sectional study (“Tübingen Survey”): This study consists of 215 male and female working age (16 to 64 years) test persons. The participants were employees from one of the research institutes of the University of Tübingen, as well as other persons and family members living in the area of Tübingen. From each subject one first morning void was collected during the year 1999. At this time, all test persons had been part of the general population of Southwestern Germany.

To facilitate comparison with longitudinal study subjects, these test persons were combined into two age groups—young adults between 15 and 30 years of age and adults 40 years and older. Each group consisted of subjects from both sexes.

At the time of sampling subjects from all studies lived across Southwestern Germany. Participation in all studies was on a voluntary basis. All studies were approved by the Ethics Commission at the State Medical Chamber of Baden-Württemberg project identification code 011-05-f, date of approval 1 March 2005 and the Ethics Commission of the Faculty of Medicine of the University Hospital Tübingen, project identification codes 80/99 and 288/99, dates of approval 9 June 1999 and 7 February 2000, respectively. *t*-Test comparability calculations were performed with the “graphpadquickcals” *t*-test. 

All urine samples are first morning voids. They were collected in PVC-free urine cups. The urine samples were refrigerated and immediately transported to the laboratory where they were deep-frozen and stored at −20 °C prior to analysis. The 247 samples of the longitudinal studies were stored between 1 month and 2 years. From 215 urine samples of the cross-sectional study 77 were stored between 3 months and 2 years. Sixty-nine samples were stored about 2.4 years and 69 about 3.75 years. Following Watkins et al. [23] and Wittassek et al. [24], we measure metabolite concentrations by standardized volume rather than based on creatinine content. The metabolites used as standards such as MEHP, mono(n-octyl) phthalate (MOP), 5OH-MEHP, 5oxo-MEHP and 5cx-MEPP were synthesized at the Institute of Occupational and Social Medicine, University Hospital Tübingen working in collaboration with the University of Florence [25] and the University of Essen. MnBP was purchased from Chemos (Regenstauf, Germany). Following Mettang [26,27], urine samples were incubated with 50 units β-glucuronidase prior to the extraction to ensure enzymatic hydrolysis of the glucuronidated phthalate metabolites. The samples were acidified to pH 2–3 and extracted twice with ethylacetate. After drying and concentrating, the extracts were treated successively with diazomethane and BSTFA (*N*,*O*-*bis*(trimethylsilyl) trifluoroacetamide). The quantitative analysis was performed by selected ion monitoring (at *m*/*z* 148) gas chromatography/mass spectrometry (GC-MS) operating the mass spectrometer in a negative ion chemical ionization mode [26,27]. A 20-m capillary column (DB1, 0.18 mm inner diameter, 0.18 µm film thickness (J&W Scientific, Langen, Germany) was used. The column oven temperature was programmed as follows: 40 °C (1 min), 20 °C/min to 80 °C, 10°/min to 200 °C, 5 °C/min to 280 °C (5 min). Hydrogen was used as carrier gas (40 kPa). Quality of analysis was confirmed by external controls. In this set-up, the detection limit of MnBP, MEHP, 5OH-MEHP, 5oxo-MEHP, and cx-MEPP was 0.1 μg/L urine.

## 3. Results and Discussion

### 3.1. Di(2-ethylhexyl) Phthalate (DEHP) and Di-n-butyl Phthalate (DnBP)—Metabolites in Morning Urine

In a first overview the results of metabolites of DEHP and DnBP concentrations in morning urine from both longitudinal studies as well as the cross-sectional “Tübingen Survey” are presented in Table 1. As can be seen there, concentrations seem to differ substantially across age group, sex and living conditions. For instance, median values of 5OH-MEHP concentrations for female probands range from 17.5 µg/L for students from the boarding school up to 65.6 µg/L for female subjects in age group from 15 to 30 years from the 4 × 7 days (“course of the year”) study. The corresponding values of the 95th percentile in these two groups reach 58.9 µg/L and 426 µg/L. For 5cx-MEPP, we measure median concentrations between 47.0 µg/L among males aged 40 years or older from the 4 × 7 days (“course of the year”) study and 82.0 µg/L for males from the same study who fall into the age group between 15 and 30 years. Respectively the 95th percentiles take values of 443 µg/L in the older sub-sample and 493 µg/L in the younger (see Table 1). In summary, findings are characterized by a large dispersion of metabolite concentrations across individuals.

**Figure 1:**
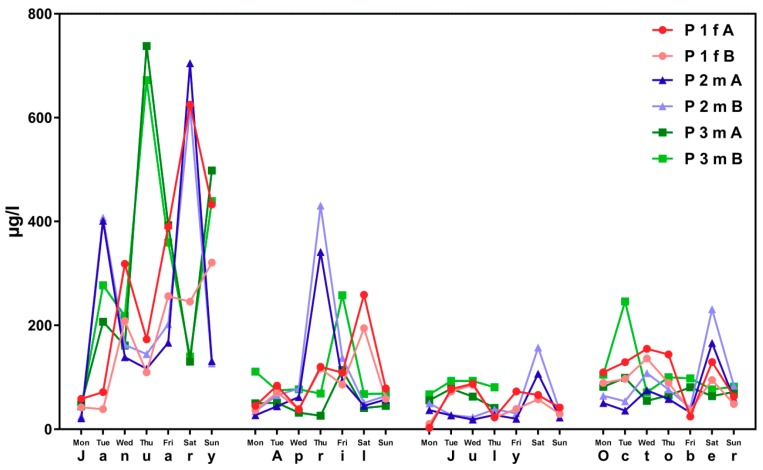
Variability in urinary concentrations of ∑5OH-MEHP + 5oxo-MEHP (A) and 5cx-MEPP (B) in morning urine of one female (f) and two male (m) subjects at the age between 15 and 30 years (general population) over four periods of seven days each (one per season of the year). The number starting with “P” is the subject identifier.

This result becomes even apparent when considering the change in ∑(5OH-MEHP + 5oxo-MEHP) and 5cx-MEPP concentrations over time. Figure 1 shows inter-day variability of metabolite concentrations over the examination period for subjects aged 15 to 30 years from the 4 × 7 days (“course of the year”) longitudinal study.

**Figure 2:**
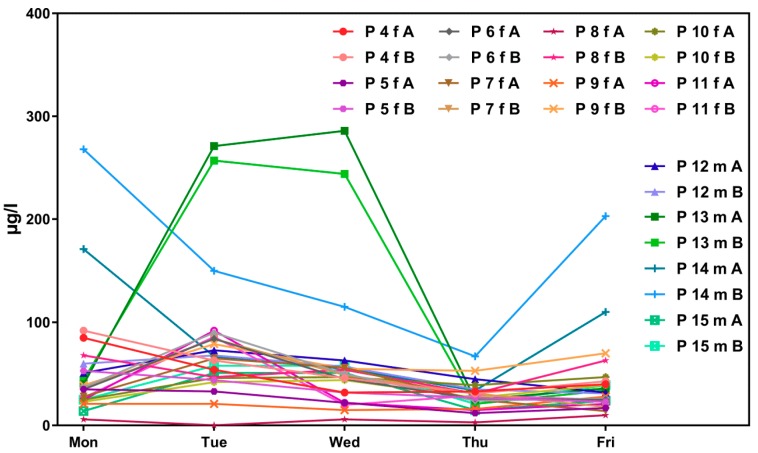
Variability in urinary concentrations of ∑5OH-MEHP + 5oxo-MEHP (A) and 5cx-MEPP (B) in morning urine of eight female (f) and four male (m) subjects at the age between 17 and 20 years (students of a boarding school) over the course of a school week. The number starting with “P” is the subject identifier.

**Figure 3:**
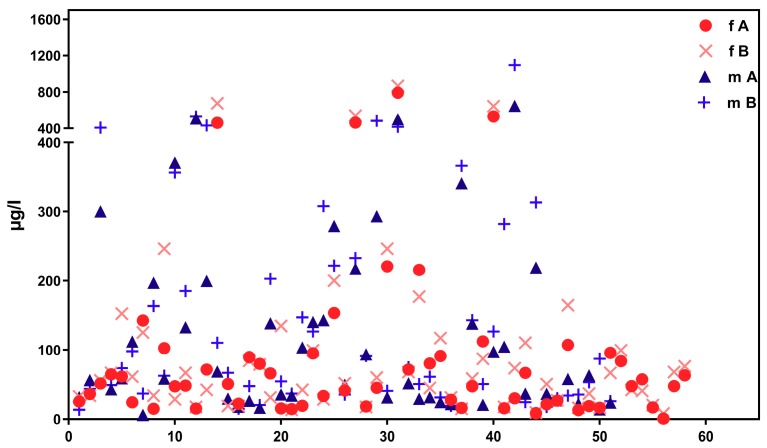
Concentration distribution of ∑5OH-MEHP + 5oxo-MEHP (A) und 5cx-MEPP (B) in morning urine of 58 female (f, red symbols) and 51 male (m, blue symbols). Subjects aged between 15 and 30 years from the general population (“Tübingen Survey”). X-axis: urine samples (from female and male participants) in arbitrary order.

**Figure 4:**
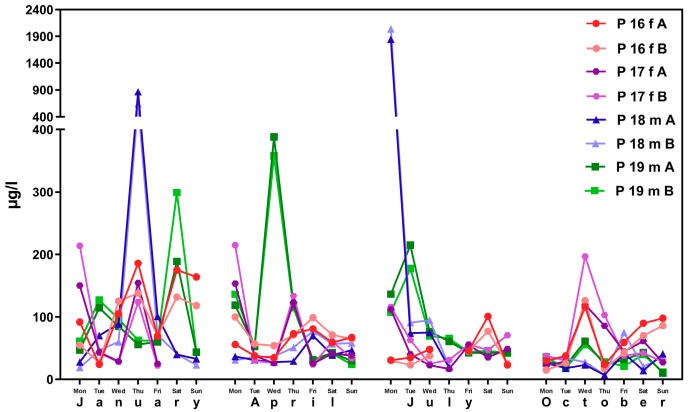
Variability in urinary-concentrations of ∑5OH-MEHP + 5oxo-MEHP (A) and 5cx-MEPP (B) concentrations in morning urine of two female (f) and two male (m) subjects aged 40 years or older (general population) over four periods of seven days each (one per season of the year). The number starting with “P” is the subject identifier.

**Figure 5:**
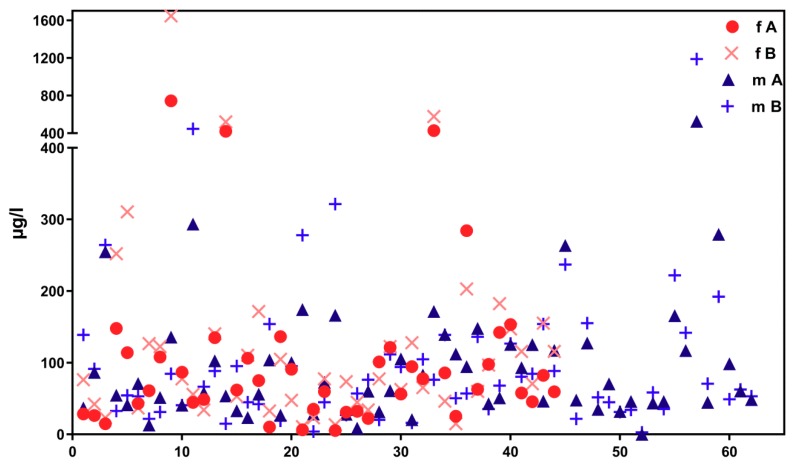
Concentration distribution of ∑5OH-MEHP + 5oxo-MEHP (A) und 5cx-MEPP (B) in morning urine of 44 female (f, red symbols) and 62 male (m, blue symbols). Subjects aged 40 years or older from the general population (“Tübingen Survey”). X-axis: urine samples (from female and male participants) in arbitrary order.

Figure 2 shows corresponding results for the sub-sample of boarding school students in a similar age group (“course of the week“-longitudinal study). The main difference between the two groups is the degree of homogeneity in living conditions, which is higher among boarding school students. Figure 3 gives the results for participants aged between 15 and 30 obtained with the “Tübingen Survey” cross-sectional study. Finally, Figure 4 and Figure 5 give results for older participants (≥40 years) obtained from the 4 × 7 days longitudinal study and the “Tübingen Survey“.

### 3.2. Comparability of the Individual Inter-Day Variability of Phthalate Metabolites in Urine Obtained by Longitudinal Studies with the Results of the “Tübingen Survey” Cross-Sectional Study

**Figure 6:**
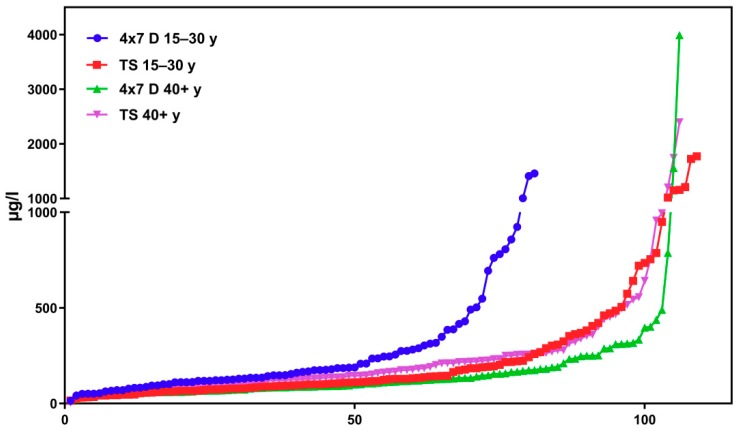
Results for the sum of MEHP + 5OH-MEHP + 5oxo-MEHP + 5cx-MEPP in the morning urine in ascending order. All test subjects age >15 years. Comparison of longitudinal and cross-sectional studies. 4 × 7 d = course of the year study; TS = “Tübingen Survey” y = age in years. X-axis: urine samples in corresponding order.

**Figure 7:**
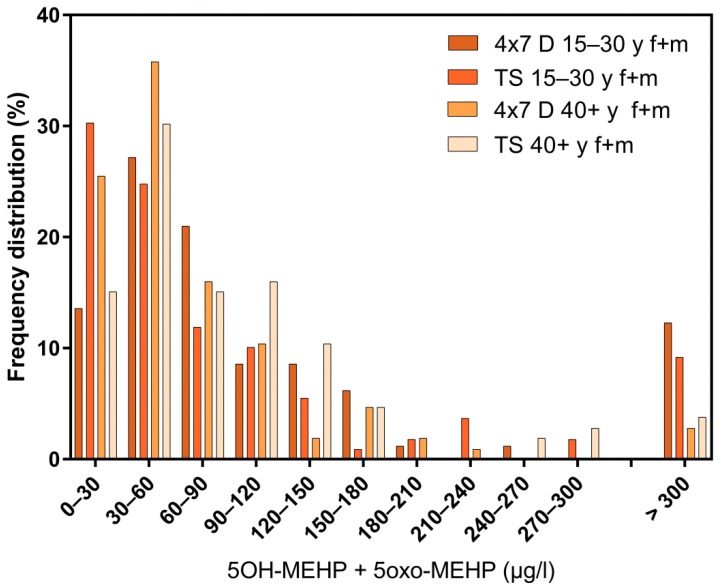
Frequency distribution of the sum of 5OH-MEHP + 5oxo-MEHP in the morning urine of female (f) and male (m) participants. All test subjects age >15 years. Comparison of longitudinal and cross-sectional studies; 4 × 7 d = course of the year study; TS = “Tübingen Survey”; y = age in years. X-axis: categories for ∑5OH-MEHP+5oxo-MEHP in µg/L in morning urine.

Regarding the results of classical cross-sectional studies one of the essential characteristics of the exposure to DEHP and DnBP is a large variation in the metabolite urinary concentration across test subjects [2,11,12,13,14,15,16,17,23,24,25,26,27,28,29,30,31,32]. Table 1 and Figure 3 and Figure 5 confirm this statement for the results of the “Tübingen Survey” presented here. Table 1 as well as Figure 1, Figure 2 and Figure 4 indicate a first hint that the distribution of the concentration of urinary DEHP metabolites obtained from few participants over a longer period of time is not significantly different from the concentration levels of many subjects at a single time point. This raises the question of whether long-term studies with few subjects lead to comparable results with the results of cross-sectional studies. When examining the results more closely, either by comparing all obtained results of a certain subject group in ascending order (compare Figure 6) or calculating the distribution frequency (compare Figure 7), it can be shown that the overwhelming majority of individual samples of all subjects falls into a comparatively narrow bandwidth of metabolite exposure with low concentrations. Observations with concentrations of metabolites of DEHP beyond this range are rare (compare also Figure 6 and Figure 7). The similar frequency distribution for the sum of 5OH-MEHP + 5oxo-MEHP shown in Figure 7 is a clear indicator for the comparability of results obtained by the course of the year - longitudinal study with the present cross-sectional study. 

In contrast, only the results for female students of the boarding school show a significantly smaller variation of metabolite concentration in urine (compare Figure 2 and Table 2). Further, two more detailed possibilities to assess possible differences between results obtained from longitudinal studies compared to results obtained from cross-sectional-studies are discussed. One is given by determining *p*-values with the *t*-test to show significant differences between two data rows based on means and standard deviations. In Table 2 means and standard deviations for all subject groups are presented. The results of *t*-testing show, that based on the sum of 5OH-MEHP and 5oxo-MEHP only the results for female and male students of the boarding school (*p* = 0.0015; confidence interval 95%) and the results for female subjects of the “Tübingen Survey” of the age group ≥40 years versus the results for female subjects of the course of the year study differ significantly (*p* = 0.0396; confidence interval 95%). The comparison of the results of all other subjects groups prove that there is no significant difference between the results obtained from longitudinal versus cross-sectional studies. 

In regard to MnBP in urine, the results are less clear. Significant differences can be detected by comparing the results of both age groups of male participants of the “Tübingen Survey” with the results of the course of the year study (*p* = 0.0185; confidence interval 95% for age group 15–30 years; *p* = 0.0005 for age group ≥40 years; confidence interval 95%). Significant differences can also be detected by comparing the results for female and male participants, age group ≥40 years of the course of the year study (*p* = 0.0046; confidence interval 95%) and by comparing the results for female participants of the course of the year study with the participants of “Tübingen Survey” regarding the age group ≥40 years (*p* = 0.0414; confidence interval 95%). 

**Figure 8:**
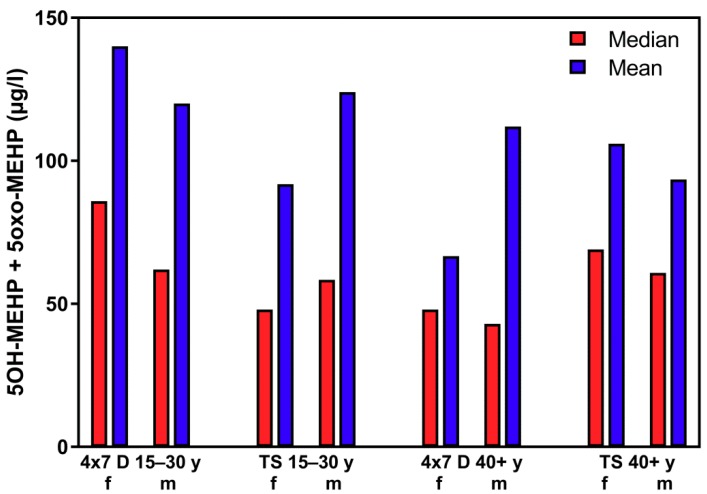
Mean and 50th percentile (median) of the sum of 5OH-MEHP + 5oxo-MEHP for female (f) and male (m) participants aged 15–30 years and ≥40 years for the “course of the years”-longitudinal study (4 × 7 d) and the “Tübingen Survey” cross-sectional study.

Figure 8 shows quite clearly, that the 50th percentile (median) and the mean of the different subject groups from the “course of the year”-longitudinal study do not differ significantly from 50th percentile (median) and the mean from the “Tübingen Survey”-cross-sectional study (compare also Table 1 and Table 2).

Another possibility to assess comparability is to consider the share of values exceeding the given guide-line values detected either by longitudinal or cross-sectional studies. In Germany Human BioMonitoring (HBM) values are implemented by the German “Human Biomonitoring Commission”. These HBM values are derived from toxicological and epidemiological studies [28]. For DEHP respectively the sum of 5OH-MEHP + 5oxo-MEHP age and gender specific different HBM I values exist [28]. These HBM values are useful to assess a higher body burden with metabolites of DEHP. Due to the age and gender specific different HBM I values female and male participants are assessed separately. For the group of female subjects of the course of a week and of the course of the year longitudinal studies four of 161 samples exceeded this HBM I value. The results of the “Tübingen Survey” show that five of 152 samples obtained from females exceeded the HBM I value. For male subjects two of 126 samples of both of the longitudinal studies exceeded HBM I while none of the samples obtained from male participants of “Tübingen Survey” exceeded HBM I.

In describing the exposure of the general population to the plasticizers DnBP and DEHP, the literature commonly uses characteristics of an empirical distribution, such as the median, the range, or the 95th percentile, of MnBP and DEHP metabolites in urine samples [2,11,12,13,14,15,16,17,23,29,30,31,32]. In the present paper, we take a longitudinal perspective by repeatedly measuring concentration values of these metabolites among a small number of subjects over time and contrast results of these studies with findings from a cross-sectional survey (see Table 1 and Table 2). We discuss whether the exposure of the general population is reflected in the results from the two longitudinal studies. A comparison must take into account a number of factors. For example, the age structure of the studies used for comparison often differs. Moreover, there has been a reduction in the burden of phthalates on the general population in recent years [12,13,14]. Another limitation of the study is perhaps, that some of the urine samples of the “Tübingen Survey”cross-sectional study were stored at −20 °C longer than two years. However, data from the literature [33] show that urine samples used for quality control are stable for at least two years.

With respect to the large variation in the metabolite urinary concentrations and the limitations mentioned above, Table 3 shows exemplary, that median, 95P and maximum for the age group of young adults (15–30 years) determined with both of the longitudinal studies in general do not differ significantly from data reported in literature for adolescents and young adults (11–21 years). 

Participants aged between 15 and 30 from the presented longitudinal studies differ in one important aspect. Test persons from the 4 × 7 days study (“course of the year”) are individuals living a normal life in Southwestern Germany. They have been randomly selected and correspond to the general population. In contrast, subjects from the 1 × 5 days study (“course of the week”) are comprised of students from a full-time boarding school, implying that this group lives, attends classes and eats together. A comparison of Figure 1 and Figure 2 (age group 15–30 years) demonstrates this influence on the metabolites of DEHP. Especially for female students the mean and standard deviation of the DEHP metabolites 5OH-MEHP, 5oxo-MEHP and 5cx-MEPP are lower among female boarding school students than among all other groups of test persons (see Table 2). *t*-Test also proves, that the results for this subject group differs significantly to results obtained from individuals living a so called “normal” life. For all other subject groups no significant difference between results for DEHP metabolites obtained in longitudinal studies to results obtained in the cross-sectional study are detectable. For MnBP we found that data obtained in longitudinal studies differ for many subject groups from results gained with the presented cross-sectional study. Thus the available data do not permit to establish a significant effect of life style on exposure to DnBP.

As noted earlier, results presented in Figure 1, Figure 2, Figure 3, Figure 4 and Figure 5 show that individual exposure to DEHP tends to be characterized by a small number of events with high loads. This finding corresponds well to concentration profiles of monoethyl phthalate (MEP) and 5OH-MEHP (respectively MEHHP) exposure reported by Preau et al. and Calafat [15,19].

In general we find that median DEHP metabolite concentrations in first morning urine voids are similar between the longitudinal studies which are based on a limited number of test persons, and the more common cross-sectional surveys if no special circumstances exist. In contrast to these surveys, however, longitudinal studies provide some indications on the frequency by which individual subjects are or were exposed to the risk of increased DEHP intake. Intra-individual sampling also suggests, that individual high urine concentrations observed in cross-sectional studies are not necessarily an indication of a health risk. The present data prove, that high concentrations of metabolites of DEHP in urine normally are rare outliers and the body burden with DEHP returns to a lower level within 1 or 2 days.

In summary, results suggest that longitudinal studies, even when based on a limited number of test persons, are a useful complement to the common cross-sectional surveys. In particular, because they provide information on the frequency by which individual members of the general population experience increased levels of DEHP exposure. Moreover, our findings highlight the importance of living conditions on these incidence rates.

## 4. Conclusions

Results from the present study show that the substantial heterogeneity in DEHP exposure characteristic for the general population, is reflected in the intra-individual variation of test persons whose exposure level is repeatedly measured over time. For DnBP the correlation of longitudinal versus cross-sectional studies is less clear. That is, when tracking individual exposure over time, the resulting series is usually characterized by low to moderate levels of intake, infrequently punctuated by high exposure events. These events are commonly short-lived such that measured loads rapidly regress to pre-event levels. As a consequence, measuring exposure to DEHP and DnBP among comparatively few individuals who are followed over time yields empirical load distributions that are comparable to the ones obtained from a larger cross-sectional sample of the same population. Nevertheless, average (median respectively mean) exposure levels can change from one population to the next, as they depend to a substantial degree on living conditions and dietary customs. Our results suggest that homogenous living conditions, such as those found in the boarding school, for example, lead to a significant influence in the variation of daily exposure to DEHP. For DnBP a comparable relation cannot be seen. Longitudinal studies thus complement the more classical cross-sectional surveys by providing information on the frequency of high-exposure events—at least during the time of examination.

## Figures and Tables

**Table 1:** Minimum, median (50th percentile), 95th percentile, maximum for urinary concentrations of MEHP, 5OH-MEHP, 5oxo-MEHP, 5cx-MEPP, MnBP stratified by study design, age and sex.

Subject Group										
Age Group	15–30 Years						≥40 Years			
Study Design	Longitudinal				Cross-Sectional		Longitudinal		Cross-Sectional	
	“Course of a Week”		“Course of the Year”				“Course of the Year”			
Study	1 × 5 Days B-School		4 × 7 Days		“Tübingen Survey”		4 × 7 Days		“Tübingen Survey”	
sex	f	m	f	m	f	m	f	m	f	m
n	40	20	28	53	58	51	53	53	44	62
P	8	4	1	2	58	51	2	2	44	62
**MEHP in µg/L in morning urine**										
Min	<1	<1	1.2	<1	<1	<1	<1	<1	<1	<1
50P	2.5	5.0	11.5	9.0	8.0	9.0	8.5	4.0	11.0	10.5
95P	13.9	18.8	50.8	61.6	36.3	44.0	42.0	44.0	141	104
Max	16.0	19.0	53.0	87.7	65.0	119	69.1	107	198	438
**5OH-MEHP in µg/L in morning urine**										
Min	<1	10.0	2.8	13.3	<1	3.4	10.3	4.0	3.4	<1
50P	17.5	35.5	65.6	41.0	26.5	35.9	31.0	31.2	42.3	42.3
95P	58.9	189	426	401	332	336	129	375	286	197
Max	63.0	190	499	523	487	520	134	1245	505	261
**5oxo-MEHP in µg/L in morning urine**										
Min	<1	2.0	<1	4.1	<1	2.7	<1	2.9	<1	<1
50P	9.0	13.5	20.3	19.7	18.0	22.0	14.4	12.9	26.3	23.3
95P	27.0	95.8	112	159	147	144	49.7	157	153	102
Max	29.0	96.0	125	215	305	168	57.0	603	240	267
**5cx-MEPP in µg/L in morning urine**										
Min	18.0	21.0	10.5	23.5	8.2	13.6	15.0	7.0	10.9	2.7
50P	42.5	58.0	78.9	82.0	57.2	73.6	55.0	47.0	77.6	67.4
95P	89.8	268	292	493	646	503	202	443	564	315
Max	92.0	268	321	672	869	1097	215	2037	1647	1189
**MnBP in µg/L in morning urine**										
Min	<1	2.0	<1	< 1	<1	<1	<1	<1	<1	<1
50P	30.5	12.0	13.5	13.0	20.0	28.0	19.0	9.0	18.0	26.5
95P	99.8	133	511	115	179	314	60.3	47.4	671.5	383
Max	129	135	886	177	615	464	64.0	54.0	1898	661

B-School = participants of a boarding school; *n* = total number of urine samples; *p* = number of test persons. 50P = 50th percentile; 95P = 95th percentile; f = female; m = male; 4 × 7 Days = course of the year study; 1 × 5 Days = course of the week study.

**Table 2:** Comparison of longitudinal and cross-sectional studies; Mean and standard deviation (Std.D) absolute (abs.) in µg/L and relative (rel.) in % for urinary concentration of 5OH-MEHP, 5oxo-MEHP, 5cx-MEPP, the sum of 5OH-MEHP + 5oxo-MEHP and MnBP stratified by study design, age and sex.

	Subject Group									
Age Group	15–30 Years						≥40 Years			
Study Design	Longitudinal				Cross-Sectional		Longitudinal		Cross-Sectional	
	“Course of a Week”		“Course of the Year”				“Course of the Year”			
Study	1 × 5 Days B-School		4 × 7 Days		“Tübingen Survey”		4 × 7 Days		“Tübingen Survey”	
sex	f	m	f	m	f	m	f	m	f	m
n	40	20	28	53	58	51	53	53	44	62
P	8	4	1	2	58	51	2	2	44	62
**5OH-MEHP in µg/L in morning urine**										
Mean	21.3	52.2	110	84.7	58.2	85.0	46.3	77.7	69.2	58.6
Std.D abs	15.2	51.7	115	113	92.7	104	34.2	185	91.4	50.7
Std.D rel %	71.5	99.0	104	134	159	123	73.9	239	132	86.5
**5oxo-MEHP in µg/L in morning urine**										
Mean	10.5	23.5	30.1	35.6	33.6	38.7	20.3	33.8	37.1	34.9
Std.D abs	6.8	26.4	28.6	43.9	50.4	41.1	13.9	88.8	43.6	39.8
Std.D rel %	65.0	112	94.8	123	150	106	68.5	262	118	114
**5cx-MEPP in µg/L in morning urine**										
Mean	45.5	92.8	97.7	143	110	158	68.7	113	148	110
Std.D abs	20.6	83.8	78.5	144	170	194	49.6	289	259	162
Std.D rel %	45.2	90.3	80.3	101	155	123	72.1	254	175	148
**∑5OH-MEHP + 5oxo-MEHP in µg/L in morning urine**										
Mean	31.8	75.7	140	120	91.8	124	66.7	112	106	93.5
Std.D abs	21.8	77.9	142	157	142	143	45.5	274	133	86.3
Std.D rel %	68.5	103	102	130	154	116	68.3	246	125	92.3
**MnBP in µg/L in morning urine**										
Mean	35.6	35.3	48.1	31.5	51.9	64.7	22.5	13.4	110	74.3
Std.D abs	30.9	38.0	165	39.1	97.7	92.8	18.6	13.3	309	122
Std.D rel %	86.9	108	343	124.1	188	143	82.3	99.0	280	165

B-School = participants of a boarding school; n = total number of urine samples; *p* = number of test persons; f = female; m = male; Std.D = standard deviation absolute (abs.) in µg/L and relative (rel.) in %; 4 × 7 Days = course of the year study; 1 × 5 Days = course of the week study.

**Table 3:** 5OH-MEHP, 5oxo-MEHP in morning urine for subjects aged 15 to 30 years. This paper results compared with published data from cross-sectional studies.

This Paper							Literature Data				
Study Design	Longitudinal				Cross-Sectional		Cross-Sectional				
Study	“Course of a Week” BS-Students		“Course of the Year” (4 × 7 Days)		“Tübingen Survey”		[13]	[31]	[32]	[14]	[12] *
	f	m	f	m	f	m					
**5OH-MEHP in µg/L in morning urine**											
50P	17.5	35.5	65.6	41.0	26.5	35.9	29.8	23.7	23.2	8.8	7.5
95P	58.9	189	426	401	332	336	317	271	67.5	56.7	18.3
Max	63.0	190	499	523	487	520	- -	- -	291	- -	28.3
**5oxo-MEHP in µg/L in morning urine**											
50P	9.0	13.5	20.3	19.7	18.0	22.0	19.5	12.9	35.2	5.7	5.6
95P	27.0	95.8	112	159	147	144	212	143	84.5	35.1	13.9
Max	29.0	96.0	125	215	305	168	- -	- -	204	- -	24.1

[12] * = data from 2011; course of the week study = 1 × 5 days; BS = boarding school; 50P = 50th percentile respectively median; 95P = 95th percentile; - - = no data available.
